# Practices regarding human Papillomavirus counseling and vaccination in head and neck cancer: a Canadian physician questionnaire

**DOI:** 10.1186/s40463-017-0237-8

**Published:** 2017-10-26

**Authors:** Scott Anderson, Andre Isaac, Caroline C. Jeffery, Joan L. Robinson, Daniela Migliarese Isaac, Christina Korownyk, Vincent L. Biron, Hadi Seikaly

**Affiliations:** 1 Division of Otolaryngology-Head and Neck Surgery, Department of Surgery, University of Alberta, 1E4.34, Walter Mackenzie Center 8440 - 112 Street, Edmonton, AB T6G 2B7 Canada; 2 Department of Pediatrics, The Stollery Children’s Hospital, Edmonton, AB Canada; 3 Department of Family Medicine, University of Alberta, Edmonton, AB Canada

**Keywords:** HPV, Head and neck cancer, Counseling practice

## Abstract

**Background:**

Human papillomavirus (HPV) has recently been implicated as a causative agent in a rapidly growing number of oropharyngeal cancers. Emerging literature supports the hypothesis that HPV vaccination may protect against HPV-related head and neck cancer (HNC) in addition to HPV-related cervical and anogenital disease. While the association between HPV infection and cervical cancer is widely understood, its relation to HNC is less well known. The purpose of this study was to better understand HPV counseling practices for infection and vaccination in relation to HNC of primary care physicians (PCPs), Obstetricians/Gynecologists (OBGYNs), and Otolaryngology - Head and Neck Surgeons (OHNSs) in Canada.

**Methods:**

A Canada-wide electronic questionnaire regarding counseling practices on HPV infection, transmission, and vaccination was designed and distributed to PCPs, OBGYNs, and OHNSs across Canada through electronic and paper-based methods. Basic Descriptive statistics were used to analyze responses.

**Results:**

In total, 337 physicians responded (239 family physicians, 51 OHNSs, 30 OBGYNs, and 17 pediatricians). Three out of four PCPs reported routine counseling of their patients regarding HPV infection, transmission, and vaccination. Among this group, 68% reported “never” or “rarely” counseling patients that HPV can cause HNC. The most commonly reported reason that PCPs cited for not counseling was a lack of knowledge. The majority of OHNSs (81%) and OBGYNs (97%) counseled patients regarding HPV infection, transmission, and vaccination. However, very few OHNSs (10%) regularly counseled patients with HPV-related HNC about HPV-related anogenital cancer. Similarly, very few OBGYNs (18%) regularly counseled patients with HPV related cervical/anogenital cancer about HPV related HNC.

**Conclusions:**

The rate of counseling on HPV infection, transmission, and vaccination in relation to HNC among PCPs is low. The most common reason is a lack of knowledge. Specialists rarely counsel patients with confirmed HPV-related cancer about other HPV-related malignancies. More research is needed on the relationship between different HPV-related cancers in order to better inform counseling practices.

## Background

Human papillomavirus (HPV) is a double stranded, non-enveloped DNA virus which has long been implicated in cervical cancer oncogenesis [1]. Recently, the high-risk HPV serotypes 16 and 18 that have long been associated with cervical and anogenital cancers have also been implicated as causative agents in a growing number of oral and oropharyngeal squamous cell carcinomas (OPSCC), a particular subset of head and neck cancer (HNC) [2–6]. The incidence of OPSCC is rapidly increasing in North America, and this is thought to be a direct result of the increasing prevalence of oncogenic HPV strains among the general population [7, 8]. This poses a significant public health concern as HPV infections are the most common sexually transmitted infections worldwide [9].

It is estimated that in Canada, 75% of sexually active males and females will contract an HPV infection in their lifetime [10]. All available HPV vaccines appear to offer long-term protection against serotypes 16 and 18 [11]. Since 2008, all Canadian provinces and territories implemented a routine HPV immunization program for school age girls [12]. As of December 2016, only Alberta, Manitoba, Ontario. Quebec, Nova Scotia, and Prince Edward Island include males in their routine HPV vaccination programs [13]. This decision is based on the cost of vaccine. However, there is evidence that vaccination of males is important in preventing HPV transmission and cervical cancer in women [14]. The failure to vaccinate males also does not take advantage of the potential importance of HPV vaccination in preventing other HPV-related cancers including anogenital and HNC, both of which can occur in men or women.

Multiple studies have reported that patients with a history of HPV-related malignancies have an increased risk of developing a second primary HPV-related cancer [3, 15–17]. However, a more recent study by Marzoukie et al [18] examining male HPV cancer patients reported no increased risk of second anogenital cancers. Many of these conflicting studies are heterogeneous and make several assumptions about their populations which make them difficult to compare. As a result, there exists debate within the literature as to whether an increased risk truly exists. However, given that the same HPV serotypes are implicated in multiple cancers, the authors argue that appropriate counseling practice should involve informing patients that are Infected with or exposed to an oncogenic HPV serotypes of all associated risks of HPV infection.

Although the importance of HPV as a causative agent in OPSCC has been increasingly appreciated over the past decade, our understanding of physician counseling practices for HPV infection, transmission, and vaccination with respect to HNC is limited. This study sought to explore this gap in the literature by shedding light on the topic from a Canadian perspective. Our objectives were twofold:

1. To explore current primary care physician (PCP) and specialist practices regarding counseling on HPV infection, transmission, and vaccination in relation to HNC.
2. To determine gaps in knowledge and/or areas where counseling practices can be improved in the practices of PCPs and those that treat patients with HPV-related cancer.

## Methods

We designed a self–administered questionnaire for distribution to Otolaryngologists – Head and Neck surgeons (OHNSs), Family Physicians, Pediatricians, and Obstetricians/Gynecologists (OBGYNs) based on previously published guidelines [19, 20] Responses from family physicians and primary care pediatricians were combined under the group “Primary Care Physicians” (PCPs). Health research ethics board approval was obtained from the University of Alberta prior to distribution (Pro00051794). The initial draft of the survey was piloted on a focus group composed of four local OHNSs, two pediatricians, and three family physicians. Feedback was sought and the questionnaire was further revised.

The questionnaire was distributed nationally via electronic means through professional association contact lists (ex: Canadian Pediatric Society, Canadian Society of Otolaryngology-Head and Neck Surgery, Canadian College of Family Physicians, and university faculty email lists). We excluded those who provided incomplete responses to the questionnaire, and any non-physician or physician that had not been in practice during the past 5 years. Information was collected anonymously using the online survey software Fluid Surveys (Ottawa, ON) which complies with Canadian privacy standards, as well as hard copy distribution from October 2014 to April 2015. The full questionnaire is included in Appendix A.

### Descriptive factors

The questionnaire was divided into two parts. Part I included questions regarding demographic information. Outcomes measured included specialty of practice, current practice setting, current province of practice, years in active practice, patient populations seen in practice, and age groups seen in practice (appendix A).

### General and specific HPV counseling practices

In Part II of the questionnaire, participants were asked to report frequency of HPV counseling related to infection, transmission, and vaccination (appendix A). Physicians were asked to report how often they counsel patients with confirmed HPV-related anogenital cancer about the risk of HNC, as well as how often they counsel patients with confirmed HPV-related HNC about the risk of anogenital cancer.

### Statistical analysis

Basic descriptive statistics were used to derive estimates for overall counseling practices for the sample. For ease of interpretation, outcomes of variables with responses ranging from never to always were combined (1 and 2 = Never to rarely, 3 = sometimes, 4 to 5 = usually to always). All analysis were conducted using IBM SPSS 22 (SPSS Inc., Chicago, IL).

## Results

**Fig. 1:**
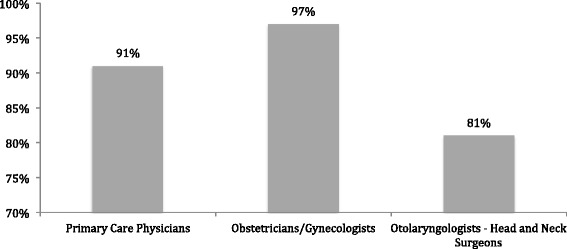
Rates of HPV infection, transmission and immunization counseling by Specialty

Characteristics of the participants are outlined in Table 1. The participants (*N* = 337) were predominantly family physicians 71%, followed by OHNSs 15%, OBGYNs 9%, and primary care Pediatricians 5% (Table 2). The majority of respondents reported practicing in a University/academic setting (58%), in which 39% were in the first 5 years of practice. The greatest number of responses came from Alberta physicians 61%, followed by those from Ontario 21%. In total, 65% of physicians saw at least as many males as females in their practice. 46%, reported counseling equally to male and female patients, while 29% reported only counseling females, and 25% counsel females more than males on the topic (Table 2). Overall, 91% of PCPs, 81% of OHNSs and 97% of OBGYNs reported that they had previously counseled a patient with regards to HPV infection, transmission, and vaccination in their practice (Fig. 1).

**Table 1:** Description of study population and characteristics

Characteristic	N = 337
Specialty of Practice	
Family Physician	71% (239)
Otolaryngologist	15% (51)
Obstetrician/Gynecologist	9% (30)
Pediatrician	5% (17)
Practice Setting	
University/academic	58% (196)
Community based	39% (131)
Other	3% (10)
Current province of practice	
Alberta	61% (206)
British Columbia	3% (10)
Manitoba	3% (10)
Nova Scotia	3% (10)
Ontario	21% (71)
Quebec	8% (27)
Saskatchewan	1% (3)
Years of active practice	
0–5	39% (131)
6–10	18% (61)
11–20	22% (74)
21–30	18% (61)
> 30	3% (10)
Patient populations seen in practice	
Only females	29% (98)
Females more than males	6% (20)
Males and females equally	64% (216)
Males more than females	1% (3)
Which age groups do you see in your practice?	
< 10 years	59%
10–14 years	68%
15–18 years	85%
19–29 years	78%
30–45 years	78%
> 45 years	80%
Which populations do you counsel regarding HPV?	
Only females	29%
Females more than males	25%
Males and females equally	46%
Which age groups do you counsel regarding HPV in your practice?	
< 10 years	33%
15–18 years	81%
19–29 years	76%
30–45	48%
> 45	14%

**Table 2:** HPV counseling topics addressed by PCPs while counseling patients

Variable	Never-Rarely	Sometimes	Usually-Always
A subset of Head and Neck cancer is caused by HPV infection	68%	10%	22%
Males are at a higher risk for HPV-related Oral and oropharyngeal cancer than females	91%	7%	2%
Sexual transmission is important in the development of HPV-related cervical cancer	1%	6%	93%
Sexual transmission is important in the development of HPV related head and neck cancer	86%	12%	2%
The HPV vaccine is effective and recommended in females	1%	3%	96%
The HPV vaccine is effective and recommended in males	20%	5%	75%
The HPV vaccine protects against the serotypes that cause cervical cancer	2%	3%	95%
The HPV vaccine protects against the serotypes that cause head and neck cancer	67%	10%	23%

When PCPs were asked to what extent they discuss that the HPV vaccine protects against HPV related cervical/anogenital cancer, 95% usually or always reported counseling on the topic (Table 2). In this same group, 67% reported never or rarely counseling that the HPV vaccine can protect against strains implicated in HPV related HNC. Furthermore, two thirds (68%) also reported “never” or “rarely” counseling that a serotype of HPV can cause HNC.

Whereas 93% of PCPs usually or always counseled patients that sexual transmission is important in the development of HPV-related cervical cancer, 86% reported never or rarely doing so with regards to risk of development of HPV-related HNC.

**Fig. 2:**
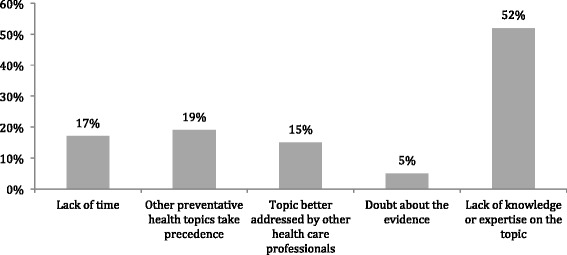
Reasons for never or rarely conducting HPV counseling related to HPV associated head and neck cancer

Overall, when asked why some physicians responded “never” or “rarely” to head and neck associated HPV counseling, the most common response was a lack of knowledge or awareness (Fig. 2).

**Fig. 3:**
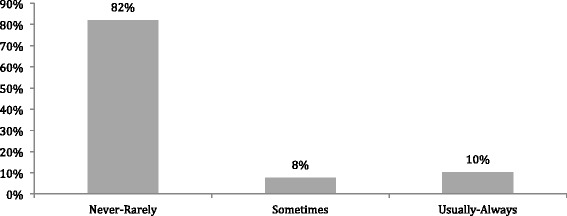
Counseling rates for patients with HPV related head and neck cancer on the risk of HPV related anogenital cancer by Otolaryngologists – Head and Neck Surgeons

**Fig. 4:**
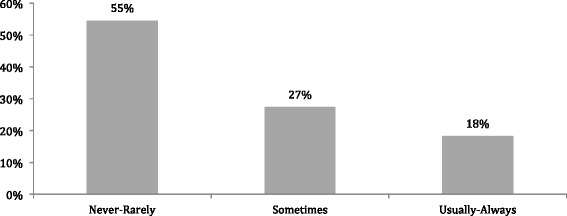
Counseling rates for patients with HPV related anogenital cancer on the risk of HPV related head and neck cancer by Obstetricians/Gynecologists

When OHNSs were asked if they counseled patients with HPV-related HNC on the potential association with anogenital cancer, 82% reported that they never or rarely do so (Fig. 3). Similarly, OBGYNs were asked to report if they counseled patients with HPV related anogenital cancer on the potential association with HPV related HNC. It was reported that 55% of Obstetrician/Gynecologist respondents never or rarely did so (Fig. 4).

## Discussion

HPV-related OPSCC has been called an epidemic in North America, owing to the increased prevalence of HPV in the general population [21]. Some predict that in the near future the rate of HPV related oral pharyngeal cancer in males may surpass the rate of cervical cancer in females in Canada [22]. Primary prevention strategies that target HPV infection, transmission, and promote vaccination have the potential to reverse this trend. PCPs, OHNSs, and OBGYNs have a central role to play in both patient education and advocacy. The development of a primary prevention strategy begins with appropriate counseling of those at risk.

This study is the first to investigate self-reported HPV counseling practices in relation to HNC in Canada. Our results suggest that while the majority of physicians do routinely counsel patients on the risks and complications associated with HPV infection, counseling on HNC is lacking.

### Primary care physicians

Among PCPs, the majority (65%) of physicians saw males in their practice setting at least as much as females. However, 54% of physicians counseled females more than males regarding HPV infection, transmission, and vaccination. These results highlight that PCPs are currently more likely to counsel patients regarding HPV if they are female rather than male. In fact, counseling of males is of critical importance in the prevention of both CSCC as well as OPSCC, as males are the primary carriers responsible for the spread of HPV infection [23].

Primary care physicians also report the most common reason as to why HPV counseling was not practiced in relation to HNC was a lack of knowledge, despite the fact that very few (5%) reported any doubt about the evidence surrounding HPV infection and its association with HNC. This may result from uncertainty with regards to the absolute benefit of HPV vaccination in HNC reduction, in addition to how PCPs prioritize HNC counseling in context of multiple competing demands and opportunities in primary care. The results highlight a specific need for further research and national or provincial professional associations with expertise in this area such as the Canadian Society of Otolaryngology-Head and Neck Surgery to educate their PCP colleagues on the changing landscape of this topic.

### Obstetricians and gynecologists

Overall OBGYNs reported a high level of patient counseling with regards to HPV infection, transmission, and vaccination (97%). This is likely due to the widely recognized relationship between HPV and cervical cancer. In fact, 100% of Gynecologist reported that when discussing the HPV vaccine they usually or always counseled patients that the vaccine protects against specific serotypes that cause cervical cancer. However, only 36% usually or always counseled that the vaccine also protects against the same subtypes that cause HPV-related HNC. The majority of OBGYNs also did not counsel patients with confirmed cervical or anogenital cancer on the association between HPV and HNC, although the authors concede that the connection between cervical cancer and HNC remains controversial.

### Otolaryngology – Head and neck surgeons

OHNSs reported similarly high rates of counseling patients with regards to HPV infection, transmission, and vaccination (81%). However, similar to OBGYNs, the OHNSs reported very low numbers for counseling patients with confirmed HPV-related HNCs on the connection between HPV and anogenital cancer (10% usually- always). The low reported levels of counseling may reflect the current debate of actual risk in the literature regarding the development of a second HPV-related malignancy. This highlights the need for more research aimed at filling this gap in order to provide guidelines for day to day practice.

Although there are no other studies with which we can compare and contrast self-reported counseling rates, it is clear that there is a paucity of literature on HPV counseling practices for HNC prevention among PCPs. There are likely multiple factors that contribute to the lack of counseling. Nevertheless it is clear that further research is required, and PCPs may benefit from knowledge support on this specific topic as the knowledge base continues to grow. Specialists may benefit from more research and recommendations for referral for additional HPV cancer screening in their patients.

Some limitations of this study included the small possibility for some selection bias by overlooking physicians who do not belong to or closely follow emails from professional societies. Also, a small sample size comprised predominantly of clinicians from Alberta, that may not be generalizable to the larger North American population. This is especially important given that different provinces have different recommendations and pubic health policies regarding HPV vaccination. Inherent in the study design (self-reported questionnaire) are a number of biases including recall bias and response bias in which there is likely error in accuracy of recall from respondents. As a result any response bias may have overinflated the true rate of counseling among physicians.

## Conclusion

The rate of counseling on HPV infection, transmission, and vaccination in relation to HNC among PCPs is low. The most common self reported reason is a lack of knowledge. Specialists rarely counsel patients with confirmed HPV-related cancer about their risk of developing other HPV-related malignancies. More research is needed on the connection between different HPV-related cancers in order to better inform counseling practices and facilitate prevention strategies.

## Abbreviations

HNC: Head and neck cancer
HPV: Human Papillomavirus
OBGYN: Obstetricians/Gynecologist
OHNS: Otolaryngology - Head and Neck Surgeon
PCPs: Primary care Physicians
SPSS: Statistical Package for the Social Science
